# Platelet-derived growth factor regulates the secretion of extracellular vesicles by adipose mesenchymal stem cells and enhances their angiogenic potential

**DOI:** 10.1186/1478-811X-12-26

**Published:** 2014-04-11

**Authors:** Tatiana Lopatina, Stefania Bruno, Ciro Tetta, Natalia Kalinina, Massimo Porta, Giovanni Camussi

**Affiliations:** 1 Department of Medical Sciences and Molecular Biotechnology Center, University of Torino, Corso Dogliotti 14, 10126, Torino, Italy; 2 EMEA LA Medical Board, Fresenius Medical CareAG & Co. KGaA, Bad Homburg 61346, Germany; 3 Department of Fundamental Medicine, Lomonosov Moscow State University, Lomonosovsky av. 31-5, 119192 Moscow, Russia

**Keywords:** Adipose mesenchymal stem cells, Extracellular vesicles, PDGF, Angiogenesis

## Abstract

**Background:**

Several studies demonstrate the role of adipose mesenchymal stem cells (ASCs) in angiogenesis. The angiogenic mechanism has been ascribed to paracrine factors since these cells secrete a plenty of signal molecules and growth factors. Recently it has been suggested that besides soluble factors, extracellular vesicles (EVs) that include exosomes and microvesicles may play a major role in cell-to-cell communication. It has been shown that EVs are implicated in the angiogenic process.

**Results:**

Herein we studied whether EVs released by ASCs may mediate the angiogenic activity of these cells. Our results demonstrated that ASC-derived EVs induced *in vitro* vessel-like structure formation by human microvascular endothelial cells (HMEC). EV-stimulated HMEC when injected subcutaneously within Matrigel in SCID mice formed vessels*.* Treatment of ASCs with platelet-derived growth factor (PDGF) stimulated the secretion of EVs, changed their protein composition and enhanced the angiogenic potential. At variance of EVs released in basal conditions, PDGF-EVs carried *c-kit* and *SCF* that played a role in angiogenesis as specific blocking antibodies inhibited *in vitro* vessel-like structure formation. The enhanced content of matrix metalloproteinases in PDGF-EVs may also account for their angiogenic activity.

**Conclusions:**

Our findings indicate that EVs released by ASCs may contribute to the ASC-induced angiogenesis and suggest that PDGF may trigger the release of EVs with an enhanced angiogenic potential.

## Background

Almost all cell types release vesicles which include exosomes and microvesicles [1-3]. Due to their heterogeneity it has been suggested to call them collectively extracellular vesicles (EVs). EVs participate in cell-to-cell communication by transfer from one cell to another proteins, bioactive lipids and nucleic acids [4,5]. EVs produced by stem cells may deliver to target cells critical information for tissue regeneration after injury. The adipose-derived stem cells (ASCs) are candidates for therapeutic application as autologous cells can be easily obtained by liposuction and expanded *in vitro*. Several beneficial effects of ASCs have been described including their ability to stimulate angiogenesis [6], nerve growth [7] and wound healing [8,9]. Recent studies indicate that ASC's regenerative actions depend on paracrine mechanisms. ASC conditioned medium contains several growth factors including vascular endothelial growth factor (VEGF) and basic fibroblast growth factor (bFGF) and may mimic the beneficial effects of cells [10,11]. We previously found that EVs released from stem/progenitor cells may, at least in part, account for the effect of conditioned medium [12]. EVs derived from endothelial progenitor cells were shown to activate an angiogenic program in quiescent endothelial cells [13].

Platelet-derived growth factor (PDGF) is crucial for the selective expansion and recruitment of undifferentiated mesenchymal cells [14-16], thus favoring wound healing and vessel formation [17-19]. PDGF induces the migration and proliferation of mural progenitor cells during vascular development [14], stimulate endothelial cells [17] and induces mesenchymal cell transdifferentiation into vessel cells [20,21].

The aim of the present study was to evaluate whether EVs derived from ASCs in basal condition (b-EVs) and after PDGF stimulation (PDGF-EVs) were able to promote angiogenesis. For these purposes, we purified EVs from non-stimulated and PDGF-stimulated ASCs and characterized their phenotype and protein content. Moreover, we evaluated the *in vitro* and *in vivo* angiogenic potential of b-EVs and PDGF-EVs.

## Results and discussion

### Characterization of ASC-derived EVs

**Figure 1:**
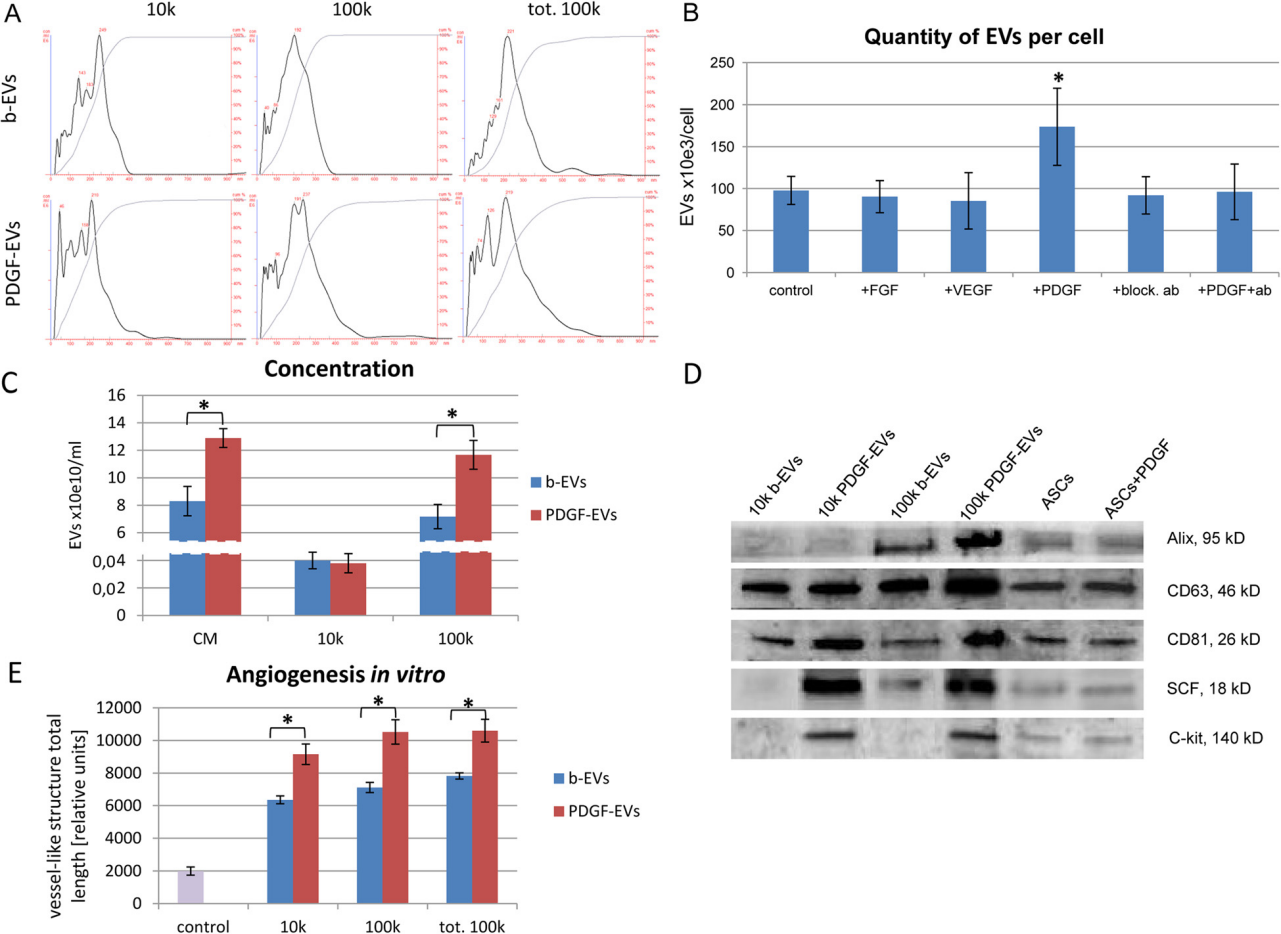
**Characterization of ASC-derived EVs subpopulations, collected after 10k and 100k g ultracentrifugation. A**: NanoSight representative images of 10k, 100k, and total 100k fractions of b-EVs and PDGF-EVs; **B**: diagram of EV quantity per single ASC after stimulation with different growth factors. Calculations of EVs were performed after ultracentrifugation of conditioned media. In selected experiments ASCs were pretreated with blocking antibodies anti PDGFRα/β and then stimulated with PDGF (+ab + PDGF), (mean ± SEM, * - p < 0,05, n = 12); **C**: diagram of concentration of EV 10k, 100k g and 100k total fractions in conditioned media of ASC (mean ± SEM, * - p < 0,05, n = 10); **D**: representative western blot analysis of exosome marker expression in the two fractions of EVs and in ASCs (10 μg of proteins/well; image of ponseau red staining in Additional file 1: Figure S1A); **E**: diagram of length of vessel-like structures after HMEC stimulation with different EV fractions; as control non-stimulated HMEC were used (mean ± SEM, * - p < 0,05, n = 5).

EVs were collected from ASC conditioned medium in basal condition and after stimulation with 20 ng/ml PDGF, FGF or VEGF. The conditioned medium was submitted to differential ultracentrifugation. After removal of cell debris and apoptotic bodies at 3k g the fraction obtained by ultracentrifugation at 10k and 100k g were analysed by NanoSight, showing similar mean size (250 ± 36 nm and 232 ± 49 nm, respectively). There was a difference between mode size of vesicles in basal conditions and after PDGF stimulation. In basal conditions mode size was unique and equal to mean size (241 ± 39); after PDGF stimulation both fractions (10k and 100k) had two mode sizes: 44 ± 6 nm and 225 ± 48 nm (Figure 1A), suggesting that PDGF stimulated the secretion of smaller EVs.

**Figure 2:**
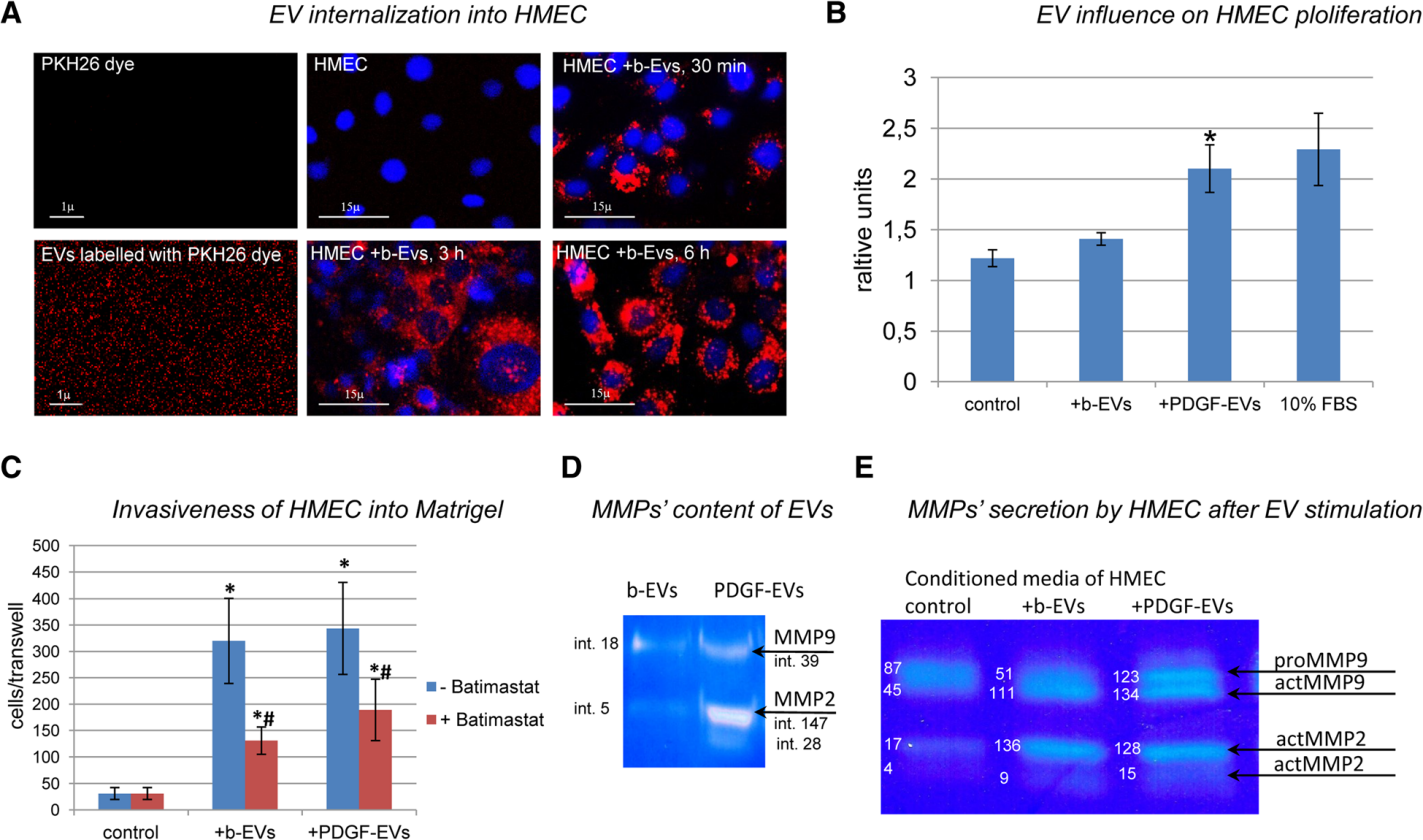
**EV effect on HMEC. A**: representative confocal microphotography of 100k total EVs labelled with PKH26 dye (red), and HMEC (nuclei are blue) incubated with EVs for 30 minutes, 3 and 6 hours; four experiments were done with similar results; **B**: diagram of HMEC proliferation in response to b-EV or PDGF-EV addition; 10% FBS was used as positive control (mean ± SEM, * p < 0.05 vs. non-stimulated control HMEC, n = 6); **C**: diagram of HMEC invasiveness into Matrigel after b-EV or PDGF-EV stimulation in presence (red column) or absence (blue column) of the MMPs inhibitor Batimastat (mean ± SEM, * p < 0.05 vs. non-stimulated HMEC, # p < 0.05 stimulation in the presence of Batimastat vs. absence of Batimastat, n = 12); **D**: representative zymography of b-EVs and PDGF-EVs; intensity of MMP2 and MMP9 bands are indicated; **E**: representative zymography of conditioned media of HMEC 24 hours after b-EV or PDGF-EV addition; proMMP-9 (92 kD), active MMP-9 (82 kD), proMMP-2 (72 kD) and active MMP-2 (62 kD) are present; the intensity (itn.) of bands is indicated.

DNA was not detected in 10k and 100k EV fractions after DNA extraction and analysis using spectrophotometry (NanoDrop) and by agarose gel electrophoresis (Bioanalyser). EVs were stained with PKH26, which labelled membrane phospholipids, indicating that particles detected both in 10K and 100K fractions were not protein aggregates (Figure 2A). Both 10k and 100k fractions expressed several mesenchymal surface markers characteristic of cell origin as seen by GUAVA FACS analysis. EVs expressed mesenchymal surface markers (CD73, CD29, CD90, CD105, CD44), endothelial markers (CD105, CD31), and marker of exosomes (CD63, CD81). The expression did not change in different fractions of EVs or EVs obtained after stimulation with PDGF with the exception of CD81 expression, which was increased in 100 k fraction of PDGF-EVs in respect to b-EVs (Table 1). Similar results were obtained by FACS analysis performed on vesicles pre-absorbed on beads (not shown).

**Table 1:** Marker expression on EV surface, shown by GUAVA FACS analysis

	**b-EVs**			**PDGF-EVs**		
	**10 k**	**100 k**	**tot.100 k**	**10 k**	**100 k**	**tot.100 k**
CD73	69,5 (±9,3)	69,5 (±8,7)	72,5 (±6,4)	72 (±9,6)	71 (±5,3)	68,5 (±4,9)
CD105	37 (±11,6)	34 (±10,0)	42,5 (±7,8)	35,5 (±8,5)	37,5 (±6,6)	50,5 (±3,5)
CD90	73,5 (±5,4)	66 (±5,3)	74,5 (±0,7)	76 (±5,0)	75,5 (±6,7)	81,5 (±2,1)
CD44	55,5 (±10,7)	53 (±7,7)	48 (±8,5)	57 (±12,8)	61,5 (±14,4)	53 (±22,6)
CD29	66 (±13,4)	59 (±8,0)	62 (±1,4)	70 (±13,9)	63,5 (±10,7)	63 (±17,0)
CD31	65,5 (±15,2)	61 (±11,8)	66,5 (±3,5)	65 (±11,6)	65,5 (±10,6)	63 (±13,1)
CD81	22 (±11,1)	**8,65 (±6,1)**	16 (±1,4)	23 (±11,2)	**21,5 (±10,5)**	22,5 (±19,1)
CD63	6,5 (±5,6)	7,5 (±5,6)	14 (±5,4)	9,25 (±5,7)	9,25 (±5,8)	25 (±7,3)

Mean ± SEM, in bold - p < 0,05, n = 4.

In basal conditions ASCs produced about 1×10^5^ ± 1×10^3^ EVs (b-EVs) per cell. As shown in Figure 1C, the amount of PDGF-EVs was significantly increased in respect to b-EVs whereas other growth factors such as FGF or VEGF did not increase EV secretion. Blocking antibodies anti PDGFR α/β inhibited the enhanced secretion of EVs by ASCs (Figure 1B). Therefore we focused on PDGF stimulation of ASCs. By NanoSight analysis the fraction of EVs increased after PDGF stimulation was the 100k fraction (Figure 1C).

The expression of CD81, CD63 and Alix was confirmed by Western blot analysis (Figure 1D).

The 10k and 100k fractions did not show significant difference in the ability to induce *in vitro* angiogenesis (Figure 1E). Taken together these experiments indicated that 10k and 100k fractions did not qualitatively and functionally differ. However, the amount of EVs obtained in the 10k fraction was 100 times lower than in 100k fraction (Figure 1C). Therefore we decided to use total 100k fraction for further experiments.

### Protein composition of ASC-derived EVs

The protein array analysis for 507 secreted proteins showed difference in protein content of b-EVs and PDGF-EVs (Table 2). After PDGF stimulation EVs expressed members of interleukin 6 group of cytokines (OSM, LIF) and their regulator sgp130, stem cell regulators such as c-kit, SCF (Figure 2B). For what concern angiogenesis, both b-EVs and PDGF-EVs contained pro- (Artemin [22], Axl [23,24], MFG-E8 [25], Osteoprotegerin [26,27]), and anti-angiogenic factors (IGFBP-7 [28], Pentraxin3 [29], sFRP-4 [30], SPARC [31-33], Thrombospondin-1 [34], TIMP-2 [35]).

**Table 2:** EV proteins that were consistently detected by protein assay

***b-EVs:***	***PDGF-EVs:***
**APRIL**	**APRIL**
Artemin*	Artemin*
Axl*	Axl*
**B7-1/CD80**	**B7-1/CD80**
**BAFF R/TNFRSF13C**	**BAFF R/TNFRSF13C**
**EDA-A2**	**EDA-A2**
**IGFBP-rp1/IGFBP-7^**	**IGFBP-rp1/IGFBP-7^**
**Kremen-2**	**Kremen-2**
**LRP-6**	**LRP-6**
MFG-E8*	MFG-E8*
**MMP-20**	**MMP-20**
Osteoprotegerin/TNFRSF11B*	Osteoprotegerin/TNFRSF11B*
**Pentraxin3/TSG-14^**	**Pentraxin3/TSG-14^**
**sFRP-4^**	**sFRP-4^**
**SPARC^**	**SPARC^**
**Thrombospondin-1^**	**Thrombospondin-1^**
**TIMP-2^**	**TIMP-2^**
Activin C	6Ckine^
Angiopoietin-like Factor*	IL-17RD
Angiostatin^	IL-20 R alpha
APJ*	Inhibin A
AR (Amphiregulin)	I-TAC/CXCL11
CCL14/HCC-1 / HCC-3	Latent TGF-beta bp1
CCL28/VIC	Lck
CV-2/Crossveinless-2	LIF^
Endostatin^	MCP-3
GCSF	MMP-10*
Glypican 3	MMP-11/Stromelysin-3
IL-1 alpha*	MMP-14
MIP 2*	MMP-9*
	OSM*
	SCF*
	SCF R/c-kit/CD117*
	sgp130
	TGF-beta 5
	Thrombopoietin (TPO)*
	TIMP-1^
	TRAIL R4/TNFRSF10D

*Indicate pro-angiogenic proteins; ^indicate anti-angiogenic proteins.

B-EVs at variance from PDGF-EVs contained pro-angiogenic factors (Angiopoietin-like Factor [36], APJ [37], IL-1α [38], MIP 2 [39]), anti-angiogenic factors Angiostatin and Endostatin [40], and polyvalent regulators Activin C [41], GCSF [42,43].

PDGF-EVs in contrast with b-EVs contained pro-angiogenic factors (Thrombopoietin [44], various types of MMPs [45], OSM [46]) and anti-angiogenic factors (6Ckine [47], TIMP-1 [48], LIF [49,50]).

B-EVs as well as PDGF-EVs did not contain PDGF; this was confirmed by Western blot analysis (not shown).

### ASC-derived EVs are internalized by HMEC, stimulate their proliferation and promote invasion

For all *in vitro* experiments we decided to use concentration of EVs equal to their concentration in ASC conditioned media. When ASCs achieved confluence the concentration was 1 × 10^11^ ± 1× 10^3^ EVs/ml that corresponded 1 × 10^5^ ± 1× 10^2^ per cell.

ASC-derived EVs entered HMEC after 30-minute incubation (Figure 2A). No difference in b-EV and PDGF-EV uptake was observed. However, PDGF-EVs significantly stimulated HMEC proliferation in respect to b-EVs (Figure 2B).

We also examined the effect of EVs on HMEC invasion. 10% of FBS was used as attractant. EVs significantly increased the invasiveness of HMEC (Figure 2C, blue columns). Since EVs contained various types of MMPs, we performed zymography analysis to investigate the mechanisms of the invasion-promoting activity of EVs. Both b-EVs and PDGF-EVs carried pro- and active forms of MMP-2 and MMP-9, but PDGF-EVs contained significantly more proMMP2 and actMMP2 (Figure 2D, Additional file 1: Figure S1C). Moreover, 24 hours after EV addition, the expression of MMPs in HMEC conditioned media was significantly increased (Figure 2E, Additional file 1: Figure S1D). Since MMPs were absent in HMEC conditioned media 4 hours after EV addition (not shown), this result cannot be accounted to the amount of MMPs present in EVs, but rather to an induction of MMPs synthesis by EV-stimulated HMEC.

Invasiveness of HMEC was partially suppressed by the addition of the MMP inhibitor Batimastat (Figure 2C, red columns) confirming the role of MMPs in EV-enhanced invasiveness of HMEC.

### ASC-derived EVs stimulated *in vitro* formation of vessel-like structures by HMEC

**Figure 3:**
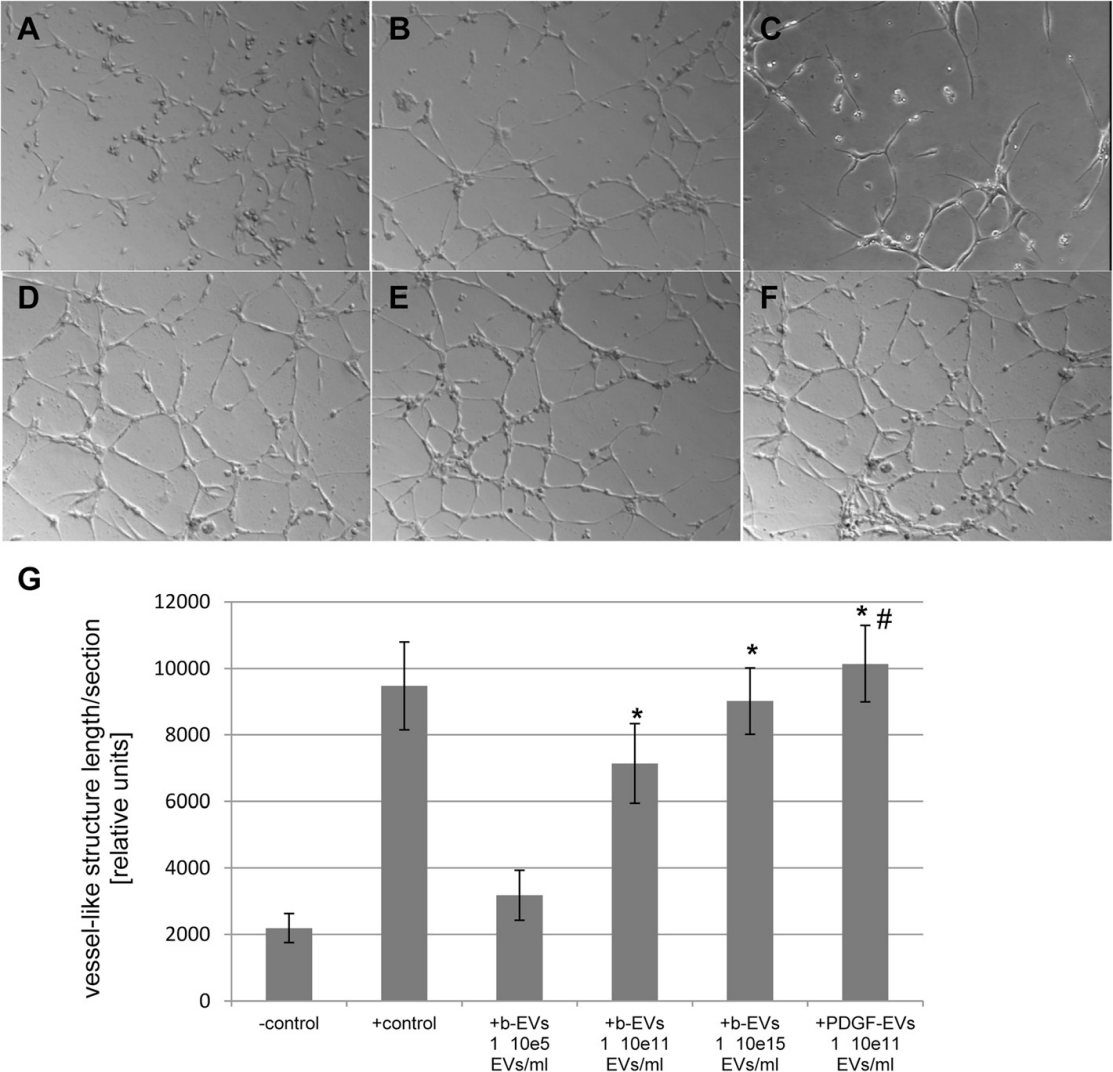
**EVs enhance the angiogenic capacity of HMEC *in vitro*.** Vessel-like structures formation by HMEC with or without EV stimulation. **A**: negative control DMEM FBS-free; **B**: positive control 20 ng/ml VEGF; **C**: b-EVs 1 × 10^5^ EVs/ml; **D**: b-EVs 1 × 10^11^ EVs/ml; **E**: b-EVs 1 × 10^15^ EVs/ml; **F**: PDGF-EVs 1 × 10^11^ EVs/ml; **G**: quantitative analysis of vessel-like structures formation in response to varies doses of b-EVs and to PDGF-EVs (mean ± SEM, * - p < 0,05 vs. “-control”, # - p < 0,05 vs. “b-EVs, 30 μg/ml”, n = 8).

EVs promoted significantly formation of vessel-like by HMEC in a dose-dependent manner, and their maximum activity (1 × 10^11^ EVs/ml) was comparable with that of VEGF (Figure 3).

The difference between stimulated HMEC and non-stimulated was evident after 48 hours, when vessel-like structures in control samples were disassembled. When compared, the *in vitro* angiogenic activity of PDGF-EVs was significantly enhanced in respect to b-EVs (Figure 3G).

To evaluate the contribution of EVs in angiogenesis we deprived the conditioned medium of EVs by ultracentrifugation and the effective deprivation was checked by NanoSight.

The difference between HMEC stimulated with EVs or EV-free supernatant was evident after 48 hours. After 48 h in EV-free supernatant samples no vessel-like structures were present, but HMEC proliferated and formed a monolayer. At 48 h EV-stimulated HMEC showed stable vessel-like structures, whereas boiling abrogated EV effects (Additional file 1: Figure S1B).

Similar results were obtained in co-culture experiments, when ASCs were placed in upper chamber of transwell: co-culture with ASCs stimulated proliferation of HMEC rather vessel formation.

### ASC-derived EVs enhance angiogenic capacity of HMEC *in vivo*

**Figure 4:**
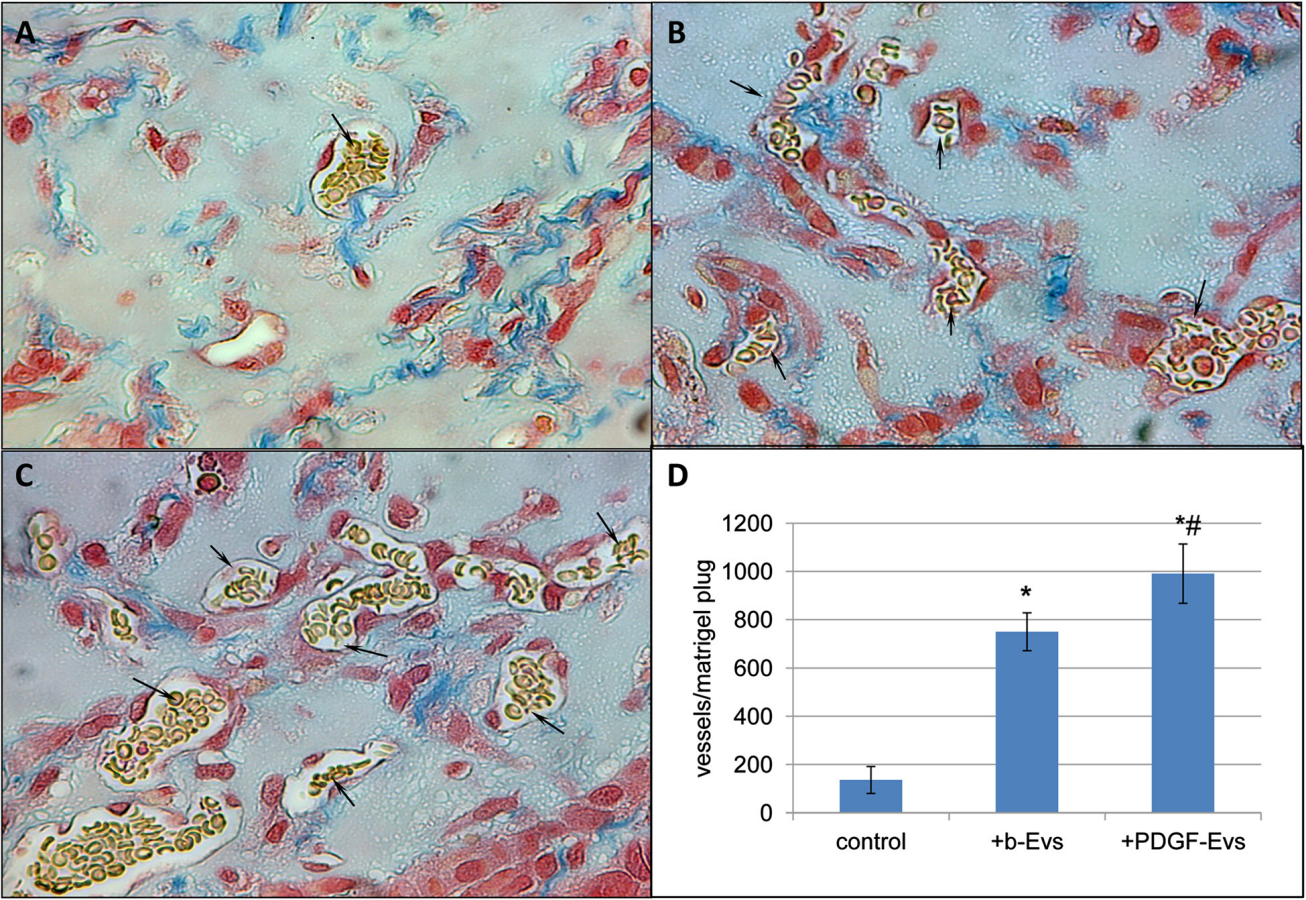
**b-EVs and PDGF-EVs stimulate angiogenesis *in vivo*. A-C**: representative Matrigel sections stained by trichrome method. Erythrocyte-contained vessels are indicated by arrows; A: spontaneous vessel formation in control Matrigel plug, containing non-stimulated HMEC; **B**: vessel formation in Matrigel plug, containing HMEC stimulated with b-EVs; **C**: vessel formation in Matrigel plug, containing HMEC stimulated with PDGF-EVs; **D**: quantitative analysis of vessel formation counted in 10 sections of Matrigel at ×20 magnification per each experimental condition; (mean ± SEM, * - p < 0,05 vs. “-control”, # - p < 0,05 vs. “b-EVs”, n = 5).

Previously HMEC were stimulated with EVs (1 × 10^10^ EVs per 1 × 10^6^ HMEC) during 3 hours at 37°C, than the cells were mixed with Matrigel (1 × 10^6^ cells/500 μl) and subcutaneously injected into SCID mice. Ten days after injection, the Matrigel plugs were excised, fixed, and embedded. As shown in Figure 4, angiogenesis was significantly enhanced by HMEC pre-stimulation with EVs (Figure 4A, B, D). PDGF-EVs significantly increased angiogenesis in respect to b-EVs (Figure 4C and D).

### c-kit/SCF signaling pathway play an important role in angiogenic activity of PDGF-EVs

**Figure 5:**
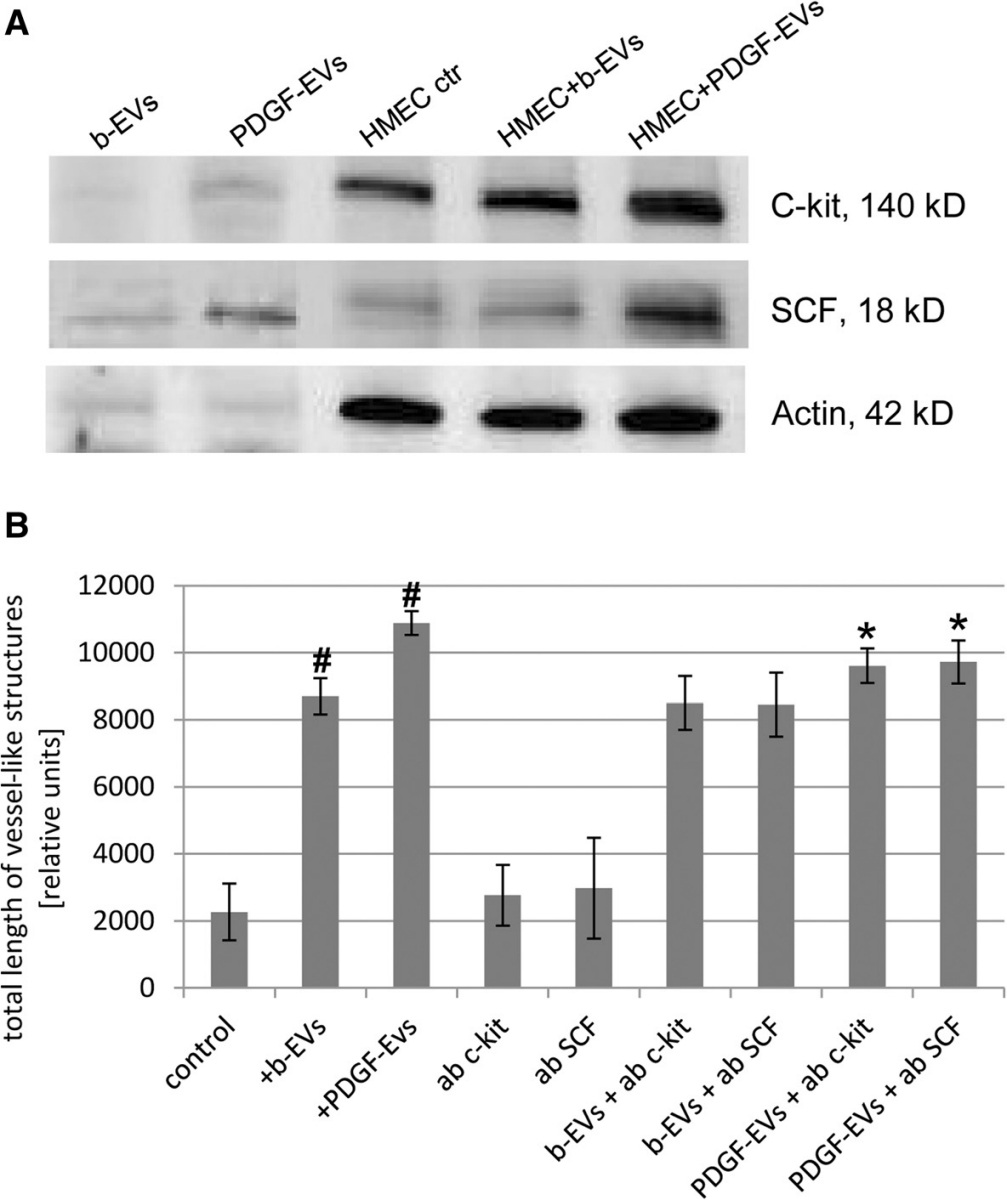
**Effect of anti-c-kit or anti-SCF blocking antibodies on *in vitro *vessel-like structure formation by HMEC. A**: representative western blot analysis of b-EVs, PDGF-EVs and HMEC after stimulation with these EVs; **B**: quantitative analysis of vessel-like structures formation in response to EVs at presence of blocking antibodies anti-c-kit or anti-SCF (mean ± SEM, # - p < 0,05 vs. “control”, * - p < 0,05 vs. “PDGF-EVs”, n = 7).

Since PDGF-EVs carry c-kit and SCF (Figure 5A) we evaluated their role in PDGF-EV-induced angiogenesis. For this purpose we incubated PDGF-EVs with neutralizing antibodies anti-c-kit or anti-SCF (at concentration 1 μg per 1 × 10^11^ EVs), then we washed PDGF-EVs with DMEM by ultracentrifugation at 100k g for 1 hour at 4°C. For controls we have used isotopic irrelevant antibody as well as anti-c-kit and anti-SCF antibodies incubated with HMEC in the absence of EVs. Incubation HMEC with PDGF-EVs changed the expression of c-kit and SCF in the cells (Figure 5A). As HMEC express c-kit and SCF, blocking antibodies interacted with endogenous c-kit and SCF and therefore abrogated their pro-angiogenic activity. Blockade of SCF or c-kit did not change action of b-EVs, but the enhanced pro-angiogenic activity of PDGF-EVs was significantly inhibited (Figure 5B), but not completely abrogated suggesting the presence of other pro-angiogenic factors.

## Conclusions

The results of the present study demonstrated that EVs released from ASCs stimulated *in vitro* and *in vivo* angiogenesis and that PDGF enhanced EV release and their angiogenic properties. Previous studies demonstrated that EVs produced by bone-marrow derived MSCs contribute to tissue remodeling after injury [12]. In the present study we focused on the angiogenic potential of EVs derived from ASCs that may represent a suitable source of adult stem cells for regenerative medicine. We found that ASC-EVs contain a set of angiogenic factors such as MFG-E8, ANGPTL1, Thrombopoietin. Moreover, EVs were found to carry MMPs that play an important role in angiogenesis by facilitating endothelial cell migration and by promoting activation of angiogenic growth factors and other signaling molecules [45]. We evaluated whether ASC stimulation with growth factors involved in angiogenesis might modify the angiogenic activity of EVs. PDGF, but not VEGF or FGF, was found to enhance EV secretion and to change their content of pro-angiogenic mediators resulting in an enhanced angiogenic activity. PDGF is known to play a key role in neovascularization after injury [17,18]. ASCs express high levels of PDGFRβ that are fundamental in the proliferation, adhesion and migration to the sites of angiogenesis [20,21,51,52]. ASCs recruited at the sites of injury may therefore contribute to angiogenesis through the release of EVs. Moreover, the finding that the protein composition of EVs was modulated by cell stimulation indicates that packing of mediators within EVs is a regulated process. Indeed, PDGF stimulated the secretion of ASC-EVs with *de novo* expression of pro-angiogenic molecules such as c-KIT and SCF, and with the absence of anti-angiogenic molecules such as Angiostatin and Endostatin. C-kit is a tyrosine kinase receptor expressed by progenitor cells differentiating into blood or vascular endothelial cells [53] and plays an important role in the amplification and mobilization of progenitor cells. Therefore, EVs carrying C-kit might recruit endothelial progenitor cells at the site of tissue remodeling. The c-kit ligand SCF promotes survival, migration, and vessel-like formation of endothelial cells and recruitment of MSCs [53,54]. The observation that blockade of c-kit and SCF significantly reduced the angiogenic potential of PDGF-EVs suggested a contribution of these factors in EV-induced angiogenesis.

In conclusion, the results of the present study demonstrate that PDGF potentiate the pro-angiogenic activity of EVs released from ASCs by enhancing their production and modulating their content of pro-angiogenic and anti-angiogenic factors.

## Methods

### Cell cultures

Human ASCs were obtained from Lonza (Basel, Switzerland), cultured in complete MSCGM™ Mesenchymal Stem Cell Growth Medium (Lonza) containing, 1% antibiotic–antimycotic (HyClone) at 37°C in 5% CO_2_ incubator. When homogeneous monolayer with typical fibroblast morphology was obtained, ASCs were passaged using trypsin solution (Sigma, St. Louis, MO, USA). For the experiments, cells of the 4–6 passages were used. At these passages phenotypic characterization and functional evaluation of mesenchymal properties were performed as previously described [55].

HMECs were purchased from Lonza (Basel, Switzerland) and were cultured in EBM-2 growth medium (Lonza) supplemented with a cocktail of angiogenic factors (SingleQuots, Lonza) according to the instructions of the manufacturer.

### Isolation and characterization of ASC-EVs

For collection of EVs from supernatants ASCs were cultured one day without FBS. In selected experiments 20 ng/ml PDGF, VEGF or FGF were added one day after deprivation. EVs were obtained from supernatants of ASCs after 2 days culture in DMEM with or without additional factors. After centrifugation at 3k g for 30 minutes to remove debris, cell-free supernatants were submitted to differential ultracentrifugation at 10k and 100k g (Beckman Coulter Optima L-90K ultracentrifuge; Beckman Coulter, Fullerton, CA) for 3 hours at 4°C. In selected experiments the 10k g centrifugation was omitted. EVs were used freshly or stored at −80°C after resuspension in DMEM supplied with 5% of DMSO. For cellular experiments frozen EVs were previously washed and pelleted by 100k g ultracentrifugation to remove DMSO. No difference in biological activity was observed between fresh and stored EVs. The protein content of MVs was quantified by Bradford method (BioRad, Hercules, CA, USA). Endotoxin contamination of MVs was excluded by Limulus test (Charles River Laboratories, Inc., Wilmington, MA, USA).

Analysis of size distribution of EVs was performed using NanoSight LM10 (NanoSight Ltd, Minton Park UK). Using a laser light source the particles in the sample are illuminated and the scattered light is captured by the camera and displayed on the connected PC running Nanoparticle Tracking Analysis (NTA). Using NTA, the particles are automatically tracked and sized based on Brownian motion and the diffusion coefficient (Dt). Results are displayed as a frequency size distribution graph and output to a spreadsheet.

To trace EVs by fluorescent microscopy, EVs were labeled with the red fluorescent aliphatic chromophore intercalating into lipid bilayers PKH26 dye (Sigma-Aldrich). After labeling, EVs were washed and ultracentrifuged at 100k g for 1 hour at 4°C. EV pellets were suspended in DMEM. Confocal microscopy was performed using a Zeiss confocal microscope, model LSM 5 PASCAL (Jena, Germany).

### Characterization of ASCs and ASC-derived EVs

Fluorescence-activated cell sorting (FACS) analysis of ASC-derived EVs was performed as described [56] using the following FITC- or PE-conjugated antibodies (Abs) directed to CD29, CD105, CD73, CD90 (Dako Cytomation, Copenhagen, Denmark), CD31 (Becton Dickinson); CD63, CD81 (Miltenyi Biotec, Bergisch Gladbach, Germany). FITC or PE mouse non-immune isotypic IgG (Dako Cytomation) was used as control.

FACS analysis of ASC-derived EVs was performed with Guava easyCyte™ Flow Cytometer (Millipore, Germany) [57,58]. To perform FACS analysis of EVs, in suspension of EVs (in quantity 250 particles per 1 μl, 100 μl) were added FITC- or PE-conjugated antibodies (mentioned previously) for 15 minutes at 4°C. Then, volume was increased with FACS flow till 500 μl. Expression of surface markers was measured according to the manufacturer’s instructions (Biocytex, France). We also performed FACS analysis after absorption on beads. Briefly, EVs (10 μg) were incubated for 30 minutes to overnight on ice with 5 μl of latex beads (Aldeyde/sulphate LATEX 4MM, invitrogen) then washed in PBS supplemented with 100 mM glycine and incubated for 30 minutes with the antibodies described above.

### DNA detection

DNA quantification was done by using Nanodrop Spectrophotometer (ND-1000, Thermo Fischer Scientific, Wilmington DE, USA), along with determining absorbance ratio at 260/280 nm for evaluating the quality of obtained viable DNA. DNA samples were also analysed using Agilent 2100 Bioanalyser protocol and the NanoChip or PicoChip. No EV DNA was detected.

### Cell proliferation assays

HMEC were seeded at 8000 cells/well into 96-well plates in DMEM deprived of FBS. B-EVs or PDGF-EVs were added (1 × 10^7^ EVs/×10^3^ cells) for 3 days. DNA synthesis was detected as incorporation of 5-bromo-2-deoxyuridine (BrdU) into the cellular DNA using an ELISA kit (Roche Applied Science, Mannheim, Germany), following the manufacturer’s instructions.

### Cell invasion assay

The effect of EVs on HMEC invasion was detected by the Transwell assay (COSTAR transwell, Corning Incorporated, MA, USA). HMEC were resuspended in DMEM (serum free) or in DMEM with b-EVs or PDGF-EVs (1 × 10^11^ EVs/ml) and were seeded into the upper compartment of an invasion chamber (30 × 10^3^ cells per well) containing a polycarbonate membrane with an 8 μm pore size which was coated with a layer of extracellular matrix (ECM; MatrigelTM, Becton Dickinson, NJ, USA). FBS (10%) was used as the attractant and added to the lower well. As a positive control, HMEC were stimulated with 10% FBS. After 48 h of incubation, the invasive cells migrated through the ECM layer to the complete medium in the lower compartment. The invasive cells were stained with Mayer and the number of invaded cells was counted. Every experiment was repeated 12 times, statistical analysis was applied among the groups.

In some experiments EVs were incubated for 1 hour at 37°C with Batimastat (1 μg/1 × 10^10^ of EVs) a MMP inhibitor and were washed by ultracentrifugation to inhibit EV-associated MMPs of before addition to HMEC.

### Gelatin zymography

Zymography was performed using “Ready Zymogram Precast Gel” (Biorad) (10%) gels copolymerized with 1 mg/ml gelatin. b-EVs and PDGF-EVs were loaded 3 μg of proteins per well. In zymogram analysis of conditioned media, media were collected from equal number of HMEC stimulated with EVs (1 × 10^11^ particle/ml). As protein concentration between different samples of media was identical, loading volume was 30 μl per well for all samples. Samples were mixed with Laemmli’s buffer without β-mercaptoethanol and incubated at room temperature for 10 min. After electrophoresis, gels were washed twice for 30 min in 2.5% Triton X-100 at room temperature, incubated overnight in collagenase buffer [50 mM Tris–HCl (pH7.5), 10 mM CaCl_2_, and 150 mM NaCl] at 37°C, and then stained with Coomassie brilliant blue R-250.

### Protein array

Purified EVs were lyzed in 1 ml of 2× Cell Lysis Buffer (RayBiotech, Inc, GA), and aliquots (1 mg of EV protein) were used for “RayBio Biotin Label-based Human Antibody Array I” (RayBiotech, Inc, GA) that was performed according to the manufacturer instructions. The biotin-conjugated antibodies on each membrane served as positive controls. The array image was captured and analyzed by the ChemiDoc™ XRS + System (Bio-Rad). The array provides detection of 507 secreted proteins. The analysis was repeated with three different samples of b-EVs and PDGF-EVs. Only proteins detected in all three experiments were mentioned as consistently detected.

### Immunoblotting

Protein samples were separated by 4% to 15% gradient sodium dodecyl sulfate–polyacrylamide gel electrophoresis and subjected to immunoblotting with antibodies to c-kit, SCF, PDGF (Abcam, UK), CD63, CD81, Alix (Santa Cruz). The protein bands were visualized with an enhanced chemiluminescence (ECL) detection kit and ChemiDoc™ XRS + System (BioRad). Cell and EV lysates were loaded at concentration of 10 μg/well. As no protein can be used as housekeeping control for concomitant detection of cells and EVs, we controlled protein transfer by ponseau staining (see Additional file 1: Figure S1A).

### Vessel-like formation assay

HMECs (30 × 10^3^ cells per well) were seeded onto Matrigel-coated wells in a 24-well plate and cultured in DMEM medium without FBS in the presence of different types of EVs in concentration 1 × 10^5^, 1 × 10^11^, 1 × 10^15^ EVs/ml. As a positive control VEGF (20 ng/ml) was used. Non-immune IgG or blocking antibody (1 μg/ml) were added when required. In experiments with EVs and blocking antibodies, EVs were washed after binding to remove unbounded antibodies. After incubation for 24 h, phase-contrast images (magnification, ×100) were recorded and the total length of the network structures was measured using MicroImage analysis system (Casti Imaging, Venice, Italy) [56]. The total length per field was calculated in five random fields and expressed as a ratio to the respective control.

### *In vivo* angiogenesis assay

Animal studies were conducted in accordance with the National Institute of Health Guide for the Care and Use of Laboratory Animals. The protocol was approved by the Committee on the Bioethics of the University of Torino (Permit Number: 1.3.10).

Angiogenesis was assayed by measuring the growth of blood vessels from subcutaneous tissue into a solid gel of basement membrane, as previously described [6,7]. Firstly, HMEC (1 × 10^6^ cells/injection) were incubated with b-EVs or PDGF-EVs (1 × 10^10^ EVs per 1 × 10^6^ of HMEC) during 3 hours. Then, male severe combined immunodeficiency (SCID) mice (8 wk old) were injected subcutaneously with 0.5 ml of ice-cold BD Matrigel Matrix Growth Factor Reduced (BD Biosciences, Franklin Lakes, NJ), which had been mixed with pre-stimulated HMEC. Equivalent quantity of non-stimulated HMEC was used as a negative control. The Matrigel plugs were excised after 10 days and fixed in 4% paraformaldehyde for 4 h. Matrigel-containing paraffin sections (5–8 μm thick) were stained by trichrome stain method [59]. The vessel lumen area (mean size per square millimeter) and quantity of erythrocyte-containing vessels were determined using computerized image analysis software MicroImage analysis system (Casti Imaging).

### Statistical analysis

All experiments were performed at least 6 times. Data was assessed for normality of distribution using the Kolmogorov-Smirnov test. Statistical analysis was performed using SigmaPlot11.0 Software. Differences between treatment and control groups were then analyzed using Student t-test. Data are expressed as mean ± SEM. We considered differences to be significant when p < 0.05.

## Abbreviations

EVs: Extracellular vesicles; ASCs: Adipose mesenchymal stem cells; HMEC: Human microvascular endothelial cells; PDGF: Platelet-derived growth factor; PDGFRα/β: Platelet-derived growth factor receptor alpha/beta; VEGF: Vascular endothelial growth factor; bFGF: Basic fibroblast growth factor; MMP: Metalloproteinase; PBS: Phosphate buffered saline; SEM: Standard error of the mean; SCF: Stem cell factor; SCID: Severe combined immunodeficiency.

## Competing interests

The authors declare that they have no competing interests.

## Authors’ contributions

TL, NK and GC developed the study idea, concept and the overall study design in addition to planning, coordinating and supervising the study. TL performed all experiments *in vitro*, TL and SB have done experiments *in vivo*. TL and GC generated the figures, wrote and edited the manuscript. MP and CT contributed to the manuscript. All authors read and approved the final manuscript.

## Supplementary Material

Additional file 1: Figure S1 **A**: representative image of ponseau red staining of western blot membrane with EVs and ASCs protein samples; **B**: quantitative analysis of vessel-like structures formation in response to co-culture with ASCs in transwell, EV-free supernatant (SuperNat), native and denatured (by boiling) EVs (mean±SEM, # - p<0,05 vs. “control”, ^ - p<0,05 vs. “b-EVs”, * - p<0,05 vs. “PDGF-EVs”, n=5); **C**: comparison of MMP expression in b-EVs and PDGF-EVs, performed by zymography and analyzed using densitometry (mean±SEM, * - p<0,05 vs. “b-EVs”, n=6); **D**: comparison of MMP expression in conditioned media of HMEC, stimulated with b-EVs or PDGF-EVs (mean±SEM, * - p<0,05 vs. “control”, n=7). Click here for file
