# Supplementary figures and images for: Platelet-derived growth factor regulates the secretion of extracellular vesicles by adipose mesenchymal stem cells and enhances their angiogenic potential
Source: Cell Commun Signal. 2014 Apr 11;12:26. doi: 10.1186/1478-811X-12-26 (PMC4022079; doi:10.1186/1478-811X-12-26)

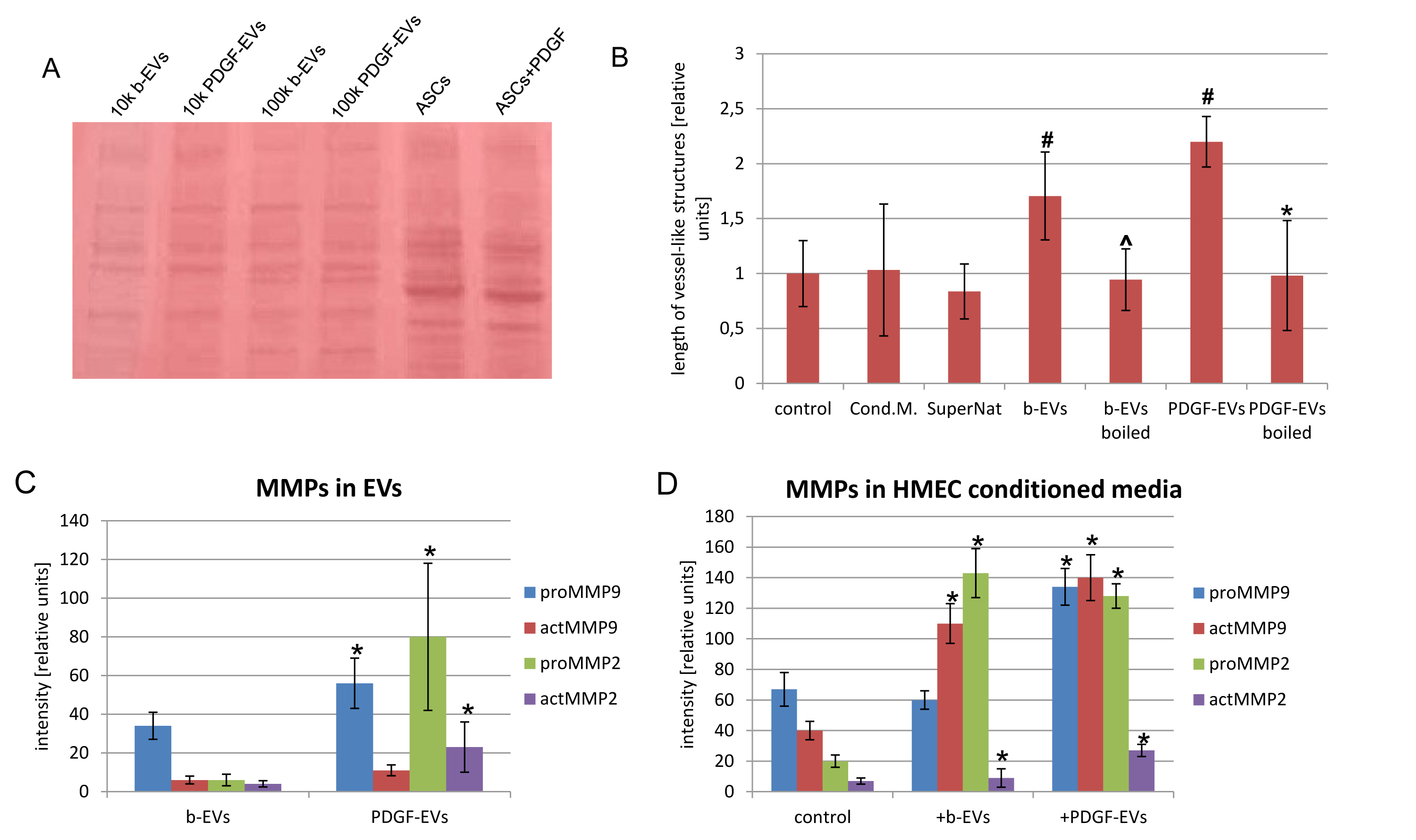

Supplement: Additional file 1: Figure S1 — A: representative image of ponseau red staining of western blot membrane with EVs and ASCs protein samples; B: quantitative analysis of vessel-like structures formation in response to co-culture with ASCs in transwell, EV-free supernatant (SuperNat), native and denatured (by boiling) EVs (mean±SEM, # - p<0,05 vs. “control”, ^ - p<0,05 vs. “b-EVs”, * - p<0,05 vs. “PDGF-EVs”, n=5); C: comparison of MMP expression in b-EVs and PDGF-EVs, performed by zymography and analyzed using densitometry (mean±SEM, * - p<0,05 vs. “b-EVs”, n=6); D: comparison of MMP expression in conditioned media of HMEC, stimulated with b-EVs or PDGF-EVs (mean±SEM, * - p<0,05 vs. “control”, n=7). [file 1478-811X-12-26-S1.tiff]
